# Editorial: Transition to adulthood in Turner syndrome

**DOI:** 10.3389/fendo.2024.1431972

**Published:** 2024-05-27

**Authors:** Małgorzata Więcek, Malcolm Donaldson, Aneta Gawlik-Starzyk

**Affiliations:** ^1^ Department of Pediatrics and Pediatric Endocrinology, School of Medicine in Katowice, Medical University of Silesia, Katowice, Poland; ^2^ Glasgow University School of Medicine, Glasgow, United Kingdom

**Keywords:** Turner syndrome, transition, puberty induction, fertility preservation, multidisciplinary approach

Turner syndrome (TS), defined as loss or abnormality of the second X chromosome, occurs in approximately 1:2000 – 1:2500 live-born female infants. Cardinal features are short stature, primary ovarian insufficiency, characteristic dysmorphic traits, and a range of associated abnormalities including congenital cardiovascular defects. Additionally, there is an increased risk of autoimmune diseases (e.g. autoimmune thyroiditis and coeliac disease). Other problems include renal anomalies, otological disease with hearing loss, visual problems, and mental health issues. Patients with TS therefore require a multidisciplinary approach with a team including endocrinologists, gynecologists, cardiologists, otolaryngologists and psychologists (1). Almost all patients require hormonal replacement therapy (HRT) to maintain satisfactory sex development and bone health (2, 3). Key issues encountered in adulthood, such as infertility, should be taken into consideration much earlier in life, to prepare the TS patient for further consequences and consider early strategies for fertility preservation (4).

For this Research Topic *Frontiers in Endocrinology* we have collected nine publications aimed at expanding our understanding of the health problems encountered in TS. This includes focus on a particularly critical phase – transition from pediatric to adult healthcare.

Improving the transition from pediatric to adult healthcare is crucial for effectively managing many chronic conditions, including TS. In this Research Topic, Zahra et al. investigated outcomes following transfer from the TS transition clinic in Glasgow to adult care. Their findings from 3- and 5-year follow-up were disappointing, with 44% of patients lost to follow-up at 5 years. This may be partly attributable to patients seeking care in other specialty clinics such as cardiology and otorhinolaryngology. The authors also highlight uncertainty as to whether these young women are receiving the recommended monitoring, hormonal replacement, and surveillance recommended.

Regarding short stature management, starting growth hormone (GH) therapy early yields the best outcomes in achieving adult height (AH). Another critical factor influencing AH is the timing of HRT for puberty induction. Kriström et al. examined AH in 132 patients with TS in relation to GH doses, age at GH and HRT. A clear dose-dependent effect on AH was found, and authors showed that starting GH treatment earlier permitted pubertal induction at a normal age. This important study underscores the benefit of starting high dose GH treatment - 67 µg/kg/day – at an early age, to maximize prepubertal height gain and normalize childhood growth.

The spectrum of primary ovarian insufficiency from adolescence until menopause, and its impact on both puberty and fertility is discussed by Porcu et al. Most individuals will require HRT to promote pubertal development, menstruation, uterine growth, and bone health. In some patients, ovarian function is preserved but tends to diminish over the lifespan, usually during adolescence. It is during this critical period when the ovaries are still functioning optimally, that fertility preservation should be considered. Factors predicting ovarian reserve include mosaic karyotype (45,X/46,XX), spontaneous puberty, gonadotropin and anti-Müllerian hormone (AMH) levels. Related to this, an article by Hagen et al. broadly addresses AMH and other factors related to puberty and fertility in TS. The authors present the protocol of *The Danish Turner Cryopreservation (DANTE) Study* and propose criteria for classifying patients for fertility preservation.

The average lifespan of individuals with TS is reduced, primarily due to a higher prevalence of congenital heart defects, arterial hypertension, aortic dissection, and metabolic disturbances including impaired glucose-insulin economy. Mitsch et al. conducted a review article on hyperglycemia in TS, exploring the factors contributing to elevated glucose levels among affected women. An early underlying deficiency in insulin secretion appears to be the leading determinant of heightened glucose levels in TS patients. Various potential causes were examined, including therapy with GH, HRT and oxandrolone; obesity, age, family history, hypogonadism, 45,X monosomy, autoimmunity, and disruption in insulin/glucagon/secretin secretion. However, consensus on the primary cause of impaired carbohydrate metabolism in this group remains elusive. Further examination of hyperglycemia and other metabolic syndrome components during GH therapy can be found in the work by Błaszczyk et al. These authors emphasize that insulin resistance and disturbances in carbohydrate metabolism are most pronounced during GH therapy in girls with TS. However, GH therapy does not seem to affect factors such as obesity, abdominal obesity, triglyceride levels, HDL concentrations, or hypertension. As metabolic disturbances can occur in women with TS throughout their lifespan, regular monitoring following transition to adult healthcare is required.

Ensuring optimal health in patients with TS necessitates adherence to the latest guidelines. Lam et al.‘s study assesses this, drawing on data from 68 patients. Recommendations for documentation of height, weight, BMI, cardiac, and renal imaging exhibited the highest implementation rates. Conversely, recommendations for bone mineral density assessment, skin examination, otological review, ophthalmological assessment, and dental consultations were implemented less frequently. Moreover, liver function biomarkers were frequently overlooked.

Recent findings from two studies underscore the variability of symptoms based on genotype. Witkowska-Krawczak et al. illustrate how different karyotypes may be associated with different healthcare needs. For instance, individuals with complete 45,X monosomy display more prominent phenotypic characteristics, a higher prevalence of congenital circulatory system abnormalities, requires HRT more frequently and show lower spontaneous menstruation compared to those with mosaicism. The study also identified a higher incidence of autoimmunity linked to the X isochromosome. Suntharalingham et al. conducted a comprehensive investigation, utilizing whole exome sequencing in 134 adult women with TS compared with 23 46,XX controls, 101 46,XX women with primary ovarian insufficiency, and 11 46,XY controls. There were no significant changes observed at the gene or variant level on the X chromosome in women with 45,X monosomy with a specific autoimmune condition compared to those without it, nor were any changes found more frequently in women without a certain condition compared to those with it. However, the authors were able to confirm a correlation between autosomal TIMP3 variation and congenital cardiac anomalies.

In conclusion, the management of TS demands comprehensive and tailored healthcare approaches, including structured transition to adult care, which address the diverse needs of patients throughout their lifespan. From optimizing growth and hormone therapies to managing metabolic and cardiovascular risks, ongoing research and clinical efforts are essential for improving outcomes in TS care.

## Author contributions

MW: Writing – original draft. MD: Writing – review & editing. AG: Writing – review & editing.
